# Thermal Acclimation of Heart Rates in Reptilian Embryos

**DOI:** 10.1371/journal.pone.0015308

**Published:** 2010-12-14

**Authors:** Wei-Guo Du, Hua Ye, Bo Zhao, Daniel A. Warner, Richard Shine

**Affiliations:** 1 Hangzhou Key Laboratory for Animal Science and Technology, Hangzhou Normal University, Hangzhou, People's Republic of China; 2 School of Biological Sciences, University of Sydney, Sydney, New South Wales, Australia; 3 Department of Ecology, Evolution and Organismal Biology, Iowa State University, Ames, Iowa, United States of America; 4 Key Laboratory of Animal Ecology and Conservational Biology, Institute of Zoology, Chinese Academy of Sciences, Beijing, People's Republic of China; Institut Pluridisciplinaire Hubert Curien, France

## Abstract

In many reptiles, the thermal regimes experienced by eggs in natural nests vary as a function of ambient weather and location, and this variation has important impacts on patterns of embryonic development. Recent advances in non-invasive measurement of embryonic heart rates allow us to answer a long-standing puzzle in reptilian developmental biology: Do the metabolic and developmental rates of embryos acclimate to local incubation regimes, as occurs for metabolic acclimation by post-hatching reptiles? Based on a strong correlation between embryonic heart rate and oxygen consumption, we used heart rates as a measure of metabolic rate. We demonstrate acclimation of heart rates relative to temperature in embryos of one turtle, one snake and one lizard species that oviposit in relatively deep nests, but found no acclimation in another lizard species that uses shallow (and hence, highly thermally variable) nests. Embryonic thermal acclimation thus is widespread, but not ubiquitous, within reptiles.

## Introduction

Most organisms inhabit thermally variable environments, and hence must cope with large temperature fluctuations over diel and seasonal cycles, coupled with marked spatial variation (e.g., between sun-exposed vs shady patches). Additionally, most organisms have little physiological control over their own body temperatures, so that much of this ambient thermal variation generates corresponding variation in body temperatures. The thermal dependence in rates of many fitness-relevant activities (e.g., locomotion, predator detection, digestion, metabolism) has imposed selection for the ability to flexibly modify physiological responses to temperature, in the frequent situation where an organism cannot control its temperature by behavioural means [1]. One widespread mechanism for buffering the physiological consequences of ambient thermal variation involves acclimation, often expressed as an ability to maintain relative constancy in important physiological processes over a wide range of thermal conditions [2], [3].

Thermal acclimation of physiological traits has been extensively reported in the post-embryonic stages of animals [2], [4]. Many physiological traits show geographic and seasonal acclimatization in nature, as well as shorter-term thermal acclimation in the laboratory. The exact form of the acclimation response differs among taxa and environmental parameters, plausibly enhancing organismal performance, and hence fitness, over a broad range of thermal conditions [2], [5].

We might expect to see acclimation in the embryos of oviparous vertebrates also. These embryos cannot thermoregulate behaviourally, and they develop in nest-sites that can experience highly variable temperatures during incubation [6]. However, logistical constraints have precluded any clear answer to the question of whether or not embryonic vertebrates are capable of acclimating to the incubation conditions that they encounter. The few studies that have looked for thermal acclimation of metabolic rates in reptilian embryos have reached contrasting conclusions, even for the same study species [7], [8]. The usual measure of metabolic rate is oxygen consumption rate, a variable that is highly dependent on embryonic mass, which in turn changes rapidly during embryogenesis and cannot be measured non-destructively. Ideally, we need a measure of embryonic developmental rate and/or metabolic rate that is stable through embryogenesis so that comparisons are not confounded by differences in embryonic developmental stage. A stable and robust index of embryonic metabolic rate would permit a straightforward test of whether or not reptilian embryos exhibit thermal acclimation.

Recent technical advances provide such a measure. We can monitor heart rates of embryonic reptiles non-invasively, by using infra-red radiation to detect subtle movements (induced by embryonic heart beat) on the shell surface [9], [10]. In this study, we first verified the assumption that heart rates are highly correlated with rates of embryonic oxygen consumption by measuring both heart rates and rates of oxygen consumption of embryos at different temperatures. We then took advantage of this new methodology to test for thermal acclimation of heart rate of embryos in four reptilian species representing different phylogenetic lineages. In reptiles, the relationship between heart rate and test temperature shows very little change from early development all the way through to immediately prior to hatching when the eggs are incubated at a constant temperature [9], [11], [12], [13], unlike the situation in some birds [14], [15]. Thus, if reptilian embryos acclimate to incubation conditions, the relationship between test temperature and heart rate should differ between conspecific embryos incubated at different temperatures – regardless of the stage of embryonic development at which we make these comparisons.

## Materials and Methods

### Study species

The common snapping turtle (*Chelydra serpentina*) is a large freshwater turtle occurring throughout much of North America [16]. Freshly-laid eggs from 12 clutches were collected from field nests near Thomson, Illinois (41°96′ N, 90°10′ W) in May 2009. The eastern fence lizard, *Sceloporus undulatus* is a medium size iguanid lizard (to 80 mm snout–vent length [SVL]) from the eastern United States; we obtained laboratory-laid eggs from gravid females (n = 16) collected from Teasdale, Mississippi (34°16′ N, 90°02′ W) in May 2009. The eastern three-lined skink *Bassiana duperreyi* is a montane species (to 80 mm SVL) from southeastern Australia. In December 2008, we collected 71 freshly-laid eggs (probably laid by about 10 females, based on the mean clutch size of 7) from field nests in the Brindabella Range (148°50′E, 35° 21′S; see [17] for details). The stripe-tailed ratsnake *Elaphe taeniura* is a large (to 1800 mm SVL) oviparous colubrid snake widespread in Asia [18]. In June 2009, we obtained 71 freshly-laid eggs from nine clutches laid at a private snake ranch at Yantai, Shandong Province of China.

### Thermal acclimation of heart rates

All eggs were weighed (±0.001 g), and lizard eggs were individually incubated in 64 ml glass jars filled with moist vermiculite (-200 KPa), snake and turtle eggs were incubated at plastic boxes (135×85×30 mm, 15 eggs in each box). The jars and plastic boxes were then randomly assigned to different temperature treatments (i.e. acclimation temperature) within the range of 20–30°C (chosen to bracket typical conditions in field nests [19], [20]). We measured heart rates of embryos at approximately 50% of the way through incubation in each species (day 42 and 35 for *C. serpentina* incubated at 25°C and 30°C respectively; day 34 and 24 for *S. undulatus* at 25°C and 28°C; day 36, 22, and 15 for *B. duperreyi* incubated at 20°C, 25°C and 30°C; day 50, 35, and 27 for *E. taeniura* incubated at 22°C, 26°C and 30°C). The eggs were transferred to an incubator set at 20, 25, or 30°C for two hours prior to being placed individually on a Buddy infrared heart rate monitor placed inside the incubator to record heart rates (see Du et al. [10] for methodology).

### Oxygen consumption and heart rate of *B. duperreyi* embryos

Rates of O_2_ consumption (V̇O_2_) of 15 eggs were measured using closed system respirometry, with the eggs inside glass jars fitted with rubber stoppers and three-way stopcocks as metabolic chambers [21], [22]. The eggs were incubated at 22±7°C. On day 40 after incubation began, one egg (plus 0.1 mL of water, to ensure vapor saturation) was added to each chamber. The chambers were then sealed and placed in incubators at temperatures of 20, 25 or 30°C for 29–45 h (with shorter duration at higher temperatures, to compensate for higher rate of O_2_ consumption), such that the ΔPO_2_ in the chamber before and after the experiment was maintained at <1% (actually from 0.2 to 0.7) to avoid the potential stress of oxygen suppression on the embryos. A gas sample was taken using a sealed syringe, and introduced into an Oxzilla II differential O_2_ analyzer (Sable Systems International, Las Vegas, USA) after being drawn through Carbosorb, to absorb CO_2_ and then through Drierite to absorb H_2_O. The O_2_ analyzer was calibrated between each measurement using room air. Output from the analyzers was recorded using ExpeData software, and rates of O_2_ consumption were calculated using the equations of Vleck [22], and corrected to standard temperature and pressure. Heart rates of the eggs were measured (using the heart rate monitor) at the same three temperatures at which rates of O_2_ consumption were determined. Mean values of O_2_ consumption rates and heart rates at each temperature were used to analyze the relationship between embryonic heart rate and oxygen consumption.

All experimental procedures were approved by the Animal Care and Ethics Committee at the University of Sydney (L04/7-2007/3/4665) and Iowa State University (3-09-6695-J), and were conducted in accordance with the NIH *Guide for the Principles of Animal Care*.

### Data analysis

We used linear regression to quantify relationships between temperature and heart rate or oxygen consumption, as well as between embryonic heart rate and oxygen consumption. Repeated measures ANOVA was used to test for the effect of incubation temperatures and test temperatures on the heart rates of embryos. Means are presented ± one standard error and significance was assumed if *P*<0.05.

## Results

### The relationship between embryonic heart rate and oxygen consumption

**Figure 1:**
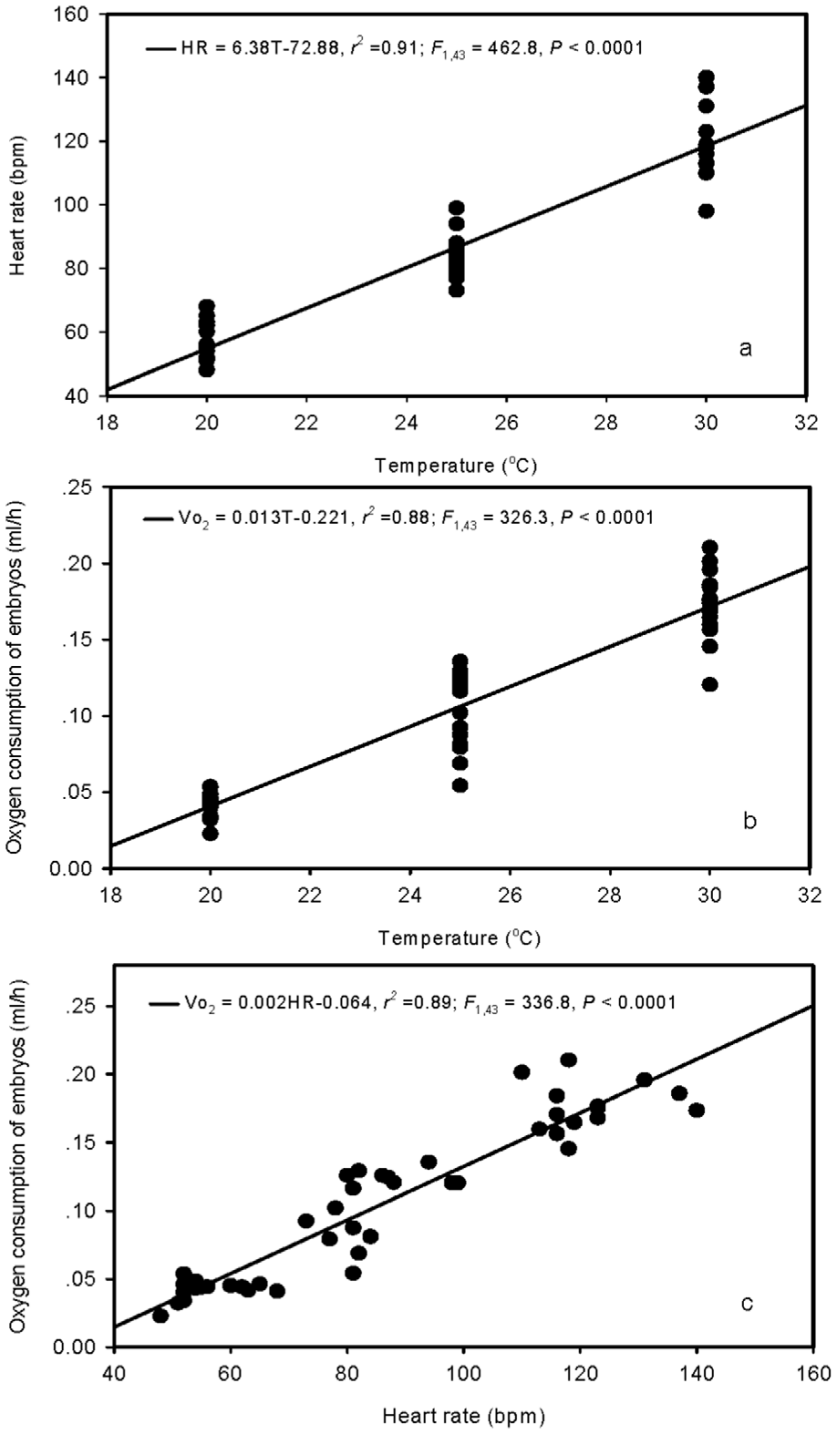
Thermal dependence of heart rate and oxygen consumption, and the relationship between embryonic heart rate and oxygen consumption in embryos of the lizard *Bassiana duperreyi*. A total of 15 eggs were incubated at 22±7°C, and their heart rates and rates of O_2_ consumption (V̇O_2_) were measured at 20, 25 and 30°C after 40 d of incubation.

Both embryonic heart rate (HR) and rate of O_2_ consumption (V̇O_2_) increased with temperature over the range of 20–30°C (HR: *F*_1,43_ = 462.8, *P*<0.0001; VO_2:_ F_1,43_ = 326.3, *P*<0.0001)(Fig. 1a,b). Embryonic heart rate was highly correlated with metabolic rate (*F*_1,43_ = 336.8, *P*<0.0001)(Fig. 1c).

### Thermal dependence and acclimation of heart rates

**Figure 2:**
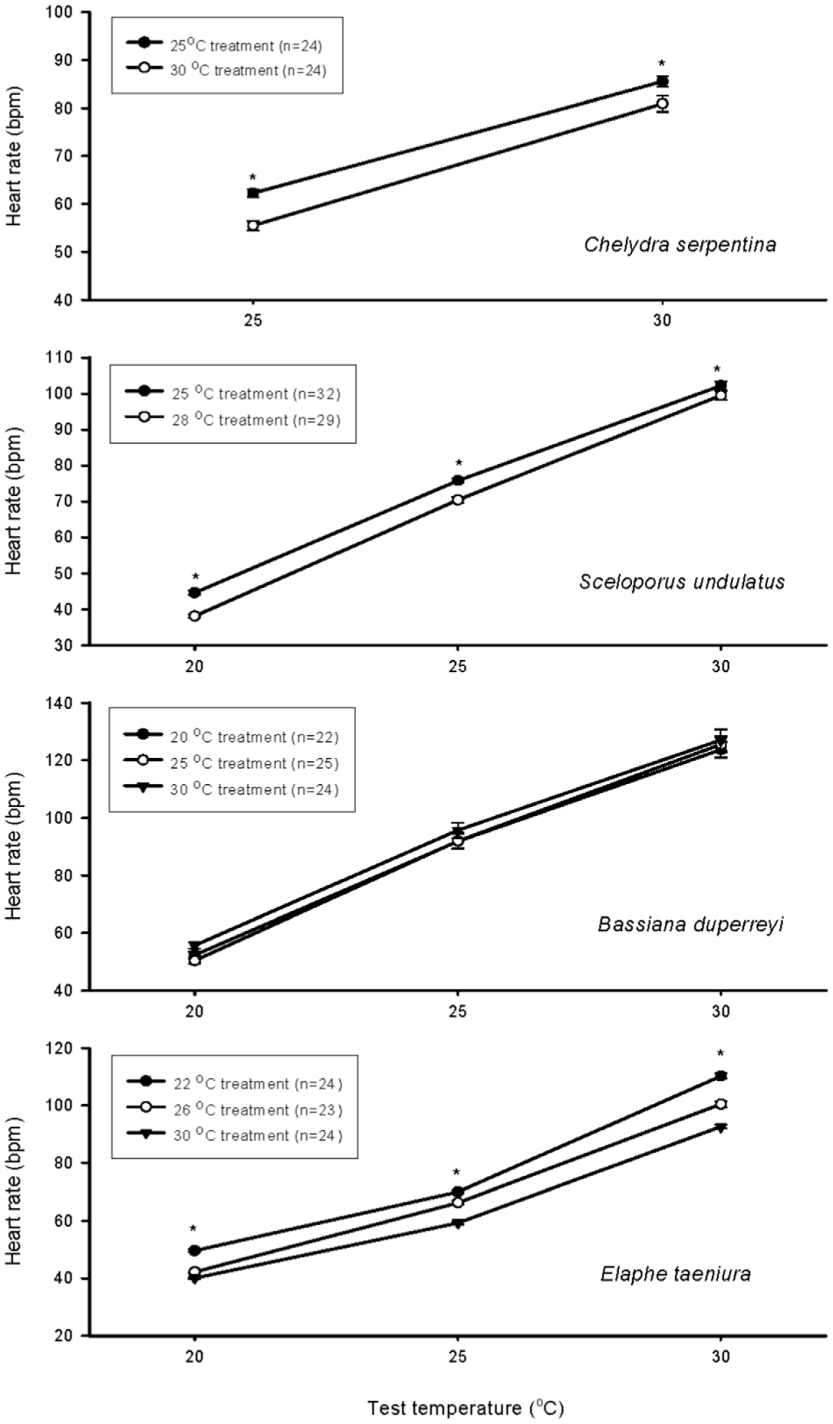
Heart rates of reptilian embryos (the turtle *Chelydra serpentina*; the lizards *Sceloporus undulatus* and *Bassiana duperreyi*; and the snake *Elaphe taeniura*) incubated at different constant temperatures. Heart rates were measured at 20, 25 or 30°C. Data are expressed as mean ± SE. Repeated measures ANOVA indicated significant thermal acclimation of embryonic heart rate in three of these species, with higher heart rates for embryos incubated at lower temperatures than those at higher temperatures. The exception was the lizard *B. duperreyi*, which showed no acclimation. Statistically significant differences between incubation temperature treatments are marked with an asterisk.

The mean egg mass was 12.70, 0.41, 0.46 and 20.36 g for *C. serpentina, S. undulatus, B. duperreyi,* and *E. taeniura,* respectively. In all four species, heart rate increased dramatically with increases in test temperature over the range of 20 to 30°C (Fig. 2). The thermal dependence of heart rate was similar in all four taxa, with coefficients linking heart rate to temperature (Q_10_) for *C. serpentina, S. undulatus, B. duperreyi,* and *E. taeniura* of 2.0, 2.4, 2.4 and 2.3 respectively.

Three of the four species also provided clear evidence of thermal acclimation of heart rates. Statistical analysis indicated that incubation temperature affected embryonic heart rate in all species except for *B. duperreyi* (Table 1). In the other three species, embryos incubated at lower temperatures had higher heart rates than conspecific embryos incubated at higher temperatures; this effect was seen at all test temperatures (Fig. 2). The coefficients (Q_10_) of temperature compensation due to thermal acclimation at test temperature of 25°C were 1.3, 1.3, and 1.2 for *C. serpentina, S. undulatus,* and *E. taeniura* (between 22 and 30°C), respectively.

**Table 1:** Results of repeated measures ANOVA for the effects of incubation and test temperatures on heart rates of embryos in four species of reptiles.

Species	Incubation temperature	Test temperature	Interaction
*Chelydra serpentina*	*F*_1,46_ = 16.84*P*<0.001	*F*_1,46_ = 601.9*P*<0.00001	*F*_1,46_ = 1.15*P* = 0.29
*Sceloporus undulatus*	*F*_1,59_ = 42.77*P*<0.00001	*F*_2,118_ = 2659.5*P*<0.00001	*F*_2,118_ = 2.88*P* = 0.06
*Bassiana duperreyi*	*F*_2,66_ = 1.03*P* = 0.36	*F*_2,132_ = 842.2*P*<0.00001	*F*_2,132_ = 0.41*P* = 0.80
*Elaphe taeniura*	*F*_2,68_ = 236.09*P*<0.00001	*F*_2,136_ = 6446.2*P*<0.00001	*F*_2,136_ = 1.72*P* = 0.10

## Discussion

An important assumption of our approach is that heart rate provides a valid index of developmental rate. Consistent with this assumption, studies on birds have indicated that fast-developing (altricial) species have a higher daily heart rate than do precocial species whose embryos develop more slowly [23]. Also, the relationship between temperature and heart rate is similar to that between temperature and metabolic rate during embryonic and larval development of fish [24], [25]. Similarly, the current study found a strong positive correlation between embryonic heart rate and oxygen consumption rate in *Bassiana duperreyi* within the temperature range of 20–30°C, suggesting that heart rate can serve as a valid index of metabolic rate. Unlike oxygen consumption rate (that is highly dependent upon developmental stage and embryonic mass), available data suggest that heart rate is relatively constant throughout most of embryonic development in reptiles when the eggs are incubated at a constant temperature [9], [11], [12], [13]. The same ontogenetic constancy is evident in our own more extensive unpublished studies of lizards (*Lampropholis delicata*), turtles (*Chrysemys picta* and *Graptemys kohnii*) and snakes (*Elaphe taeniura*). Heart rate thus appears to offer a stable and robust index of developmental rate in reptiles, at least for studies of thermal effects on rates of embryonic development.

Reflecting logistical difficulties, few previous studies have examined thermal acclimation in reptilian embryos. Studies on the turtle *Chelydra serpentina* and the crocodile *Crocodylus johnstoni* found no significant differences in metabolic rate (or heart rate) between embryos incubated at high *versus* low temperatures, suggesting metabolic compensation at the low temperature [8], [26]. Two studies on the lizard *Sceloporus undulatus* generated contrasting results, with an initial finding of metabolic acclimation [27] challenged by a later study by the same research team [7]. Our study provides explicit evidence for thermal acclimation of embryonic heart rates (Fig. 2). Incubation at low temperatures induced embryos of three out of the four species we studied, from different lineages of reptiles, to increase their heart rates (and thus metabolic rates) at any given test temperature. A similar ability to acclimate heart rates and/or metabolic rate occurs in post-hatching reptiles and fish [2], [28]. When the reptile embryos in our study were acclimated to low temperatures, the acclimation Q_10_ of their heart rates (1.2–1.3) was similar to that of heart rates in adult fish [28], showing partial compensation.

The acclimation effect results in partial compensation of metabolic and developmental rates in response to low temperatures; that is, cold conditions do not slow embryogenesis as much as they would have done in the absence of acclimation. This physiological mechanism is likely to increase egg survival and hatchling fitness, because shorter incubation periods reduce risk that an embryo will be exposed to hazards such as extreme temperatures and predation [29]. Field studies of reptiles commonly show that earlier hatching can increase the survival rate of hatchlings [30], [31], [32].

**Figure 3:**
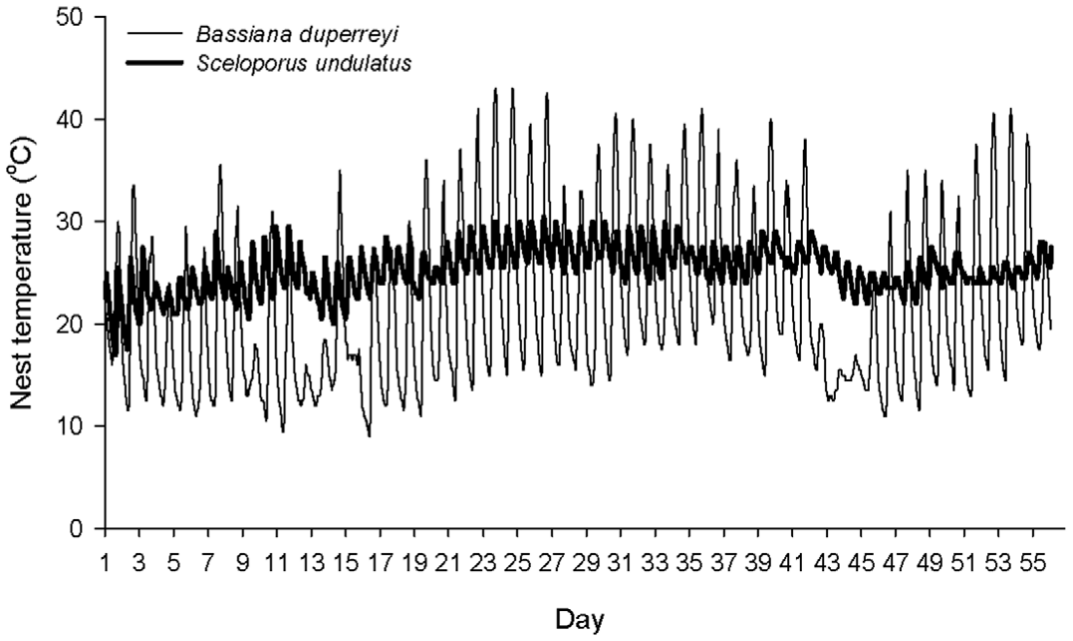
Thermal regimes within a natural nest of the lizard *Bassiana duperreyi* and an estimated nest of the lizard *Sceloporus undulatus*. Soil temperature at depth of 60 mm was used to estimate thermal regimes within a nest of *S. undulatus* with 50% canopy coverage (Angilletta et al. 2009). The data (from miniature data-loggers) span the period 8 December 2007 to 31 January 2008 in *B. duperreyi*, and 20 May to 15 July 2009 in *S. undulatus*, comprising most the incubation period in these two species. Note the stronger diel cycle in temperatures in *B. duperreyi* compared with *S. undulatus*. Thermal regimes inside snapping turtle (*Chelydra serpentina*) nests [36] also show relatively minor fluctuations compared to those of *B. duperreyi*.

Why did one of the four species that we studied (*B. duperreyi*) exhibit no thermal acclimation of heart rates? One plausible answer may involve the magnitude of diel fluctuations in nest temperature experienced by this species, compared to the others (Fig. 3). Snapping turtles, fence lizards and ratsnakes all dig relatively deep nests, where diel thermal fluctuations will be dampened because the ground above the nest buffers solar heating during the day [19], [20], [33]. In contrast, the range of *B. duperreyi* extends to the upper elevational limit for oviparous lizards in Australia, with correspondingly low mean soil temperatures [17]. The only nest-sites that offer sufficiently warm conditions for embryogenesis are shallow nests beneath rocks and logs, and temperatures within these nests often range over more than 15°C each day [17], [34]. Because physiological acclimation requires a relatively stable environmental cue [35], this diel thermal fluctuation within *B. duperreyi* nests may preclude acclimation.

Our results for the fence lizard *S. undulatus* clearly show thermal acclimation of heart rates, whereas previous studies on the same wide-ranging species have suggested that this species either does [27] or does not [7] exhibit such a response in terms of metabolic rate. We cannot explain these conflicting results, but note that the incubation temperatures (28 and 32°C) used by Angilletta et al. [7] were higher than those used in the present study (25 and 28°C). Thermal acclimation may occur only over a limited thermal range in this species; or heart rates may acclimate whereas metabolic rates do not; or (more likely, in our opinion), the newly-developed methods for monitoring of heart rates rather than oxygen consumption rates provide a more sensitive and robust way to look for acclimation effects.

In summary, our data demonstrate that acclimation of heart rate (and thus, by strong inference, developmental rate and metabolic rate [9], [10]) occurs in both turtles and squamates, but that not all species exhibit this capacity. Future work could usefully examine a wider range of species, and of conspecific populations from climatically different areas, to explore the extent to which acclimation ability is shaped by phylogenetic conservatism as compared to local adaptation. For example, inverse compensation of metabolic rates has been reported in northern populations of post-hatching individuals, apparently as an adaptation for conserving energy in winter [5]. The enormous ecological diversity of oviparous reptiles provides exciting opportunities to characterise the ways in which evolutionary forces and ecological factors shape patterns of these phenotypically plastic responses to incubation conditions.
